# Application of Design of Experiment for Polyox and Xanthan Gum Coated Floating Pulsatile Delivery of Sumatriptan Succinate in Migraine Treatment

**DOI:** 10.1155/2014/547212

**Published:** 2014-10-28

**Authors:** Swati C. Jagdale, Chandrakala R. Pawar

**Affiliations:** Department of Pharmaceutics, MAEER's Maharashtra Institute of Pharmacy, MIT Campus, Kothrud, Pune, Maharashtra 411038, India

## Abstract

Migraine follows circadian rhythm in which headache is more painful at the awakening time. This needs administration of dosage form at night time to release drug after lag period when pain gets worse. Sumatriptan succinate is a drug of choice for migraine. Sumatriptan succinate has bitter taste, low oral bioavailability, and shorter half-life. Present work deals with application of design of experiment for polyox and xanthan gum in development of press coated floating pulsatile tablet. Floating pulsatile concept was applied to increase gastric residence of the dosage form. Burst release was achieved through immediate release tablet using crospovidone as superdisintegrant (10%). Pulse lag time was achieved using swellable polymer polyox WSR 205 and xanthan gum. 3^2^ experimental design was applied. Optimized formulation was evaluated for physical characteristics and *in-vitro* and *in-vivo* study. From results, it can be concluded that optimized batch F8 containing polyox WSR205 (72.72%) and xanthan gum (27.27%) of total weight of polymer has shown floating lag time of 55 ± 2 sec, drug content of 100.35 ± 0.4%, hardness of 6 ± 0.1 Kg/cm^2^, and 98.69 ± 2% drug release in pulse manner with lag time of 7 ± 0.1 h. Optimized batch showed prolong gastric residence which was confirmed by *in-vivo* X-ray study.

## 1. Introduction

Chronopharmaceutics, the drug delivery based on circadian rhythm, is the upcoming branch of pharmacy worldwide. Various diseases like asthma, hypertension, arthritis, and migraine show circadian variation that demands time-scheduled drug release for effective drug action [1]. Floating pulsatile drug delivery system is useful for those drugs having poor oral bioavailability, low gastric residence, and narrow absorption window. Overall, these considerations led to the development of oral pulsatile release dosage forms possessing gastric retention capabilities [2]. In migraine, the risk of attacks is just before the waking hours of the patient, that is, early in the morning, and therefore the need of antimigraine is typically felt during morning hours. For such cases, conventional formulations of sumatriptan succinate cannot be administered before the symptoms get worse because at that time patients are asleep.

Sumatriptan succinate is the drug of choice for migraine and cluster headaches. It is effective in 70% of migraine attacks, but the drug has drawback of short duration of action. Sumatriptan succinate is poorly absorbed by mouth, hence giving delayed response. It does not cross blood-brain barrier and its plasma half-life is ~1.5 hours which may be due to extensive biotransformation mainly through monoamino oxidase-A. The oral bioavailability of sumatriptan succinate is 14 ± 5% owing to first pass metabolism. It has an elimination half-life of 2.5 hours and has an absorption zone from stomach to the upper part of small intestinal tract [3].

Press coated technology was used to prepare floating pulsatile tablet. In general, a press coated tablet consists of an inner core tablet and an outer coating shell. The outer layer surrounds the inner core. Selection of outer layer materials has a significant impact on the performance of the tablet, including the coating's mechanical strength, drug release characteristics, and tablet stability. Press coating may be classified as a chronopharmaceutical technology, in that it provides a solid dosage form for drug delivery in a pulsatile fashion rather than continuously with predetermined time, at the site following oral administration [4]. Also one more advantageous point taken into consideration is that press coating provides taste masking of sumatriptan succinate as drug has bitter taste thus leading to greater patient compliance regarding oral administration.

Xanthan is extracellular heteropolysaccharides produced by pure culture aerobic fermentation of carbohydrate with bacterium* xanthomonas campestris*. It is an ionic polysaccharide, whose primary structure depends on bacterial strain and fermentation condition. It is used as a tablet excipient to increase or decrease the drug release but has not much been reported concerning its use for sustained drug release. Xanthan has the potential advantage of drug release with zero order release kinetics. Muqtader used xanthan gum and guar gum to prepare floating drug delivery systems for famotidine which has higher absorption at low pH [5]. Subhash Chandra Bose et al. [6] prepared floating tablets of diltiazem HCl using xanthan gum as carrier. It was found that the formulation with low amount of xanthan gum (40% w/w) showed a low release rate compared to formulation with higher concentration (60% w/w). Radhakrishna [7] designed to formulate and evaluate balanced floating drug delivery system as controlled release module of amoxicillin trihydrate using HPMC grades and xanthan gum. Chaturvedi et al. [8] used polysaccharides and gums as reservoir for a sustained delivery of tramadol hydrochloride by hydrodynamically balanced systems.

Based on the nature of polymer and water interaction, polyethylene oxide is a hydrophilic polymer. It is a nonionic homopolymer of ethylene oxide. It is also known as polyox. Polyox is a water soluble resins and is also referred to as poly (ethylene oxide). Polyethylene oxide is used as matrix materials. Polyox water soluble resins have applications in tablet binding, tablet coatings, transdermal drug delivery systems, immediate release dosage form, and gastroretentive dosage forms. They exhibit film forming and water retention properties. It has high water solubility and low toxicity [9, 10]. Jagdale et al. [11] designed and optimized compression coated floating pulsatile drug delivery systems of bisoprolol using polyox WSR205 and polyox WSR N12K.* In vivo *study confirms burst effect at 4 h in indicating the optimization of the dosage form. Bomma and Veerabrahma [12] investigated the cefuroxime axetil sustained-release floating tablets using polymers like HPMC K4M and polymer combination of HPMC K4M and polyox WSR 303 by effervescent technique. All the formulations could sustain drug release for 12 h. Yang et al. [13] proposed asymmetric triple layer tablet for the triple drug treatment (tetracycline, metronidazole, and bismuth salt) of* Helicobacter* using HPMC and poly (ethylene oxide) as the major rate-controlling polymeric excipients. Results demonstrated that sustained delivery of tetracycline and metronidazole over 6–8 h. Mahalingam et al. [14] prepared compacts containing selected bioadhesive polymers, fillers, and binders bioadhesive gastroretentive delivery system to deliver water soluble and water insoluble compounds in the stomach. Compacts containing higher PEO showed higher swelling and bioadhesion and retained their integrity and adherence onto gastric mucosa for about 9 h under* in vitro* conditions.

Taking into consideration the pharmacokinetics as well as objective of chronotherapy of migraine, an attempt has been made to design and formulate floating pulsatile press coated drug delivery of sumatriptan succinate using polymers polyox and xanthan gum which when administered at bed time will deliver the drug in early morning hours.

## 2. Material and Methods

### 2.1. Materials

Sumatriptan succinate was obtained as a gift sample from Teva Pharmaceuticals, Goa. Polyox WSR205 was gifted by Colorcon Asia Pvt. Ltd, Mumbai, India. Other chemicals used were of analytical grade.

### 2.2. Methods

#### 2.2.1. Characterization of Drug and Excipients

Drug and excipients were characterized for color, physical appearance, and melting point and compared with standards as given in their individual profile [15]. Purity of the drug was characterized by UV analysis (Varian UV spectrophotometer) for parameters as linearity, precision, accuracy, LOD, LOQ, and robustness [16, 17]

#### 2.2.2. Compatibility Study

The study of FT-IR and DSC was used as a means for studying drug-excipients compatibility. In FT-IR study, KBr pellets were prepared with drug and excipients and scanned under Varian FT-IR in the wavelength region of 4000–400 cm^−1^. Thermal analysis was performed for drug and mixture of drug with excipients using DSC (Mettler DSC 1 star system, Zurich, Switzerland).

#### 2.2.3. Preparation of Rapid Release Core Tablet (RRCT)

All the ingredients were passed through number 60 mesh sieve (Retch) separately. The ingredients were weighed and mixed in a geometrical order. RRCTs were prepared by direct compression method. Rapid pulse release was achieved using crospovidone as superdisintegrant by varying concentration from 1 to 10%. The mixture was compressed by using 6 mm size punch to get a tablet of weight 70 mg (Cadmac, Rimeck minipress). Different trial batches were carried out as shown in Table 1.

#### 2.2.4. Preparation of the Floating Pulsatile Release Tablet (FPRT)

(*1) Preliminary Trial Batches*. At the preliminary stage, different trial batches were manufactured for floating pulsatile layer with single polymer as well as with combination of polymer polyox WSR205 and xanthan gum.

(*2) Experimental Design*. Experimental design chosen was response surface methodology and 3-level factorial was applied [18–20]. On the basis of the evaluation of trial batches, concentrations of the polymers were decided. 3^2^ full factorial designs were applied to establish the relationship in between independent (polymer ratio) and dependant variables (hardness and swelling index) using software program Design expert 8.0.7.1. In this design, 3 levels of concentrations were used and coded as −1, 0, and +1, respectively. The 3 concentrations were decided that the difference in between two consecutive levels is the same. The variations according to factorial design were carried out for three different levels of polyox WSR205 and xanthan gum is coded as shown in Table 2.

(*3) Formulation of Floating Pulsatile Tablet*. Optimized batch (RRCT-4) containing 10% concentration of superdisintegrant was used for formulation of FPRTs. Dry coating of optimized RRCT was carried out using polyox WSR205 and xanthan gum along with effervescent agent sodium bicarbonate and citric acid. Final weight of tablet was adjusted to 325 mg. Dry coated tablet was prepared by placing 50% of floating pulsatile release layer in die cavity first followed by RRCT-4 on it. Further remaining quantity of floating pulsatile release layer was added in cavity to cover the RRCT. Then, it was compressed using punch of size of 9 mm (Cadmac, Rimeck minipress). Nine batches were formulated as per the factorial design as shown in Table 3.

#### 2.2.5. Evaluation of RRCT and FPRT

(*1) Precompression Parameters*. Powder blends of RRCT as well as FPRT were evaluated for precompression parameters as angle of repose, bulk density, tapped density, Hausner's ratio, and compressibility index [21].

(*2) Postcompression Parameters*. Physical characterization, uniformity of content, and* in vitro* dissolution study were carried out for both RRCT and FPRTs according to the standard procedure [22].* In vitro* disintegrating time was carried out for RRCT. As FPRT consists of swellable polymer and effervescent agents, tablets were also evaluated for swelling index and buoyancy time. By considering all pre- and postcompression results, the optimized FPRT was studied for* in vivo* X-ray study and stability study.

*(i) Physical Characterization*. The physical characteristics, that is, thickness (Vernier caliper), diameter (Vernier caliper), hardness (Monsanto hardness tester), friability (Roche friabilator), and weight variation, were carried out.

*(ii) Uniformity of Content*. This was determined from random selection of 10 dosage units of both RRCT and FPRT using UV analytical method of sumatriptan succinate at *λ* max 227 nm.

*(iii) In Vitro Disintegrating Time*.* In vitro* disintegration time of six tablets from RRCT-4 was determined using digital tablet disintegration apparatus (Veego) at 37 ± 2°C in 900 mL 0.1 N HCl.

*(iv) In Vitro Dissolution Study*.* In vitro* dissolution studies were carried out in 0.1 N HCl (900 mL) at 37  ±  0.5°C using USP dissolution apparatus type II (Electro Lab, Mumbai) for both RRCT and FPRT. The speed of rotation was set at 50 rpm. Aliquots of dissolution medium were withdrawn for total period of 1 h in case of RRCT and at each 1 h time interval for FPRT till total disintegration of RRCT occuring after the lag period. Content of sumatriptan succinate was determined by using UV spectrophotometer at 227 nm. The dissolution studies were carried out in triplicate.

*(v) Swelling Index*. The swelling index of all factorial batches was calculated using USP dissolution apparatus type I. Six tablets were placed in basket of dissolution apparatus containing 0.1 N HCl as dissolution medium at 37 ± 0.5°C. Tablets were withdrawn at a time interval of 60 min, blotted with tissue paper to remove the excess water, and weighed on the analytical balance (Shimadzu, AUW220D). The study was conducted in triplicate. Swelling index was calculated by using the following equation [23]:

(1) Swelling  index =Wet  weight  of  tablet−Dry  weight  of  tabletDry  wet  of  tablet×100

*(vi) Buoyancy Study. In vitro* floating behavior of FPRTs was studied using dissolution apparatus type II in 900 mL 0.1 N HCl at 37 ± 0.5°C. The speed of rotation was maintained at 50 rpm. The floating lag time (the period between placing FPRT in the medium and buoyancy) and floating duration of FPRTs were determined by visual observation [24].

*(vii) In Vivo X*-*Ray Study*. The* in vivo* X-ray study for optimised batch F8 was performed on three healthy human volunteers using X-ray generating unit following guideline of world medical association. Sumatriptan succinate was replaced with radioopaque agent barium sulphate. This study was performed only to check gastric retention time for optimized delivery and it does not involve any blood or other samplings. For placebo study, volunteers aged 25-26 years and weighing 55–60 Kg were selected. The written informed consent of the human volunteers was taken before participation. The study was carried out under the supervision of an expert radiologist and physician. Prepared tablets were administered to every subject in fed condition. Radiograms were taken at 1/2, 2, 4, and 6 h [25, 26].

*(viii) Stability Study*. Accelerated stability study was carried out for optimized batch (F8) at 40 ± 2°C/75 ± 5% RH over 3-month period according to ICH guidelines in stability chamber (Thermolab India). At the end of the 3 months, the tablets were examined for physical characteristics, drug content,* in vitro* drug release (lag time), and floating lag time [27].

(*3) Statistical Analysis of Data*. Design Expert version 8.0.7.1 software program was used to establish the relationship between independent and dependant variables from obtained data of factorial batches.

The kinetics of drug release from floating pulsatile tablets was analyzed using the data obtained from the drug release studies according to the models zero order, first order, Hixson Crowell, matrix, and Korsmeyer-Peppas model. PCP Disso V3 software was used to study the kinetics of drug release.

Similarity factor study was done by using BIT software.

## 3. Results and Discussion

### 3.1. Characterization of Drug and Excipients

All the excipients and drug complied with the results of color, physical appearance, and melting point as per their individual profile. The purity of drug was confirmed by UV analysis which showed *λ* max values at 227 nm,* Y* intercept at −0.075, correlation coefficient (0.998), and slope (0.123). All other analytical parameters as precision, accuracy, and robustness had shown values of standard deviation and relative standard deviation which were found within limit (i.e., not more than 2) as specified in ICH analytical method validation guidelines. LOD and LOQ found were 0.471 *μ*g/mL and 1.428 *μ*g/mL, respectively.

### 3.2. Compatibility Study

**Figure 1:**
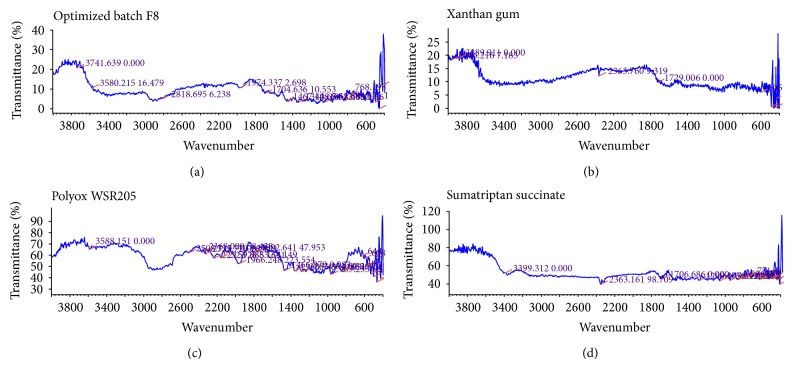
IR spectra of optimized batch F8, xanthan gum, polyox WSR205 and Sumatriptan succinate.

Characteristics peaks of sumatriptan succinate were found in the range for N–H str. primary amine at 3500–3300 cm^−1^, N–H def. at approx. 1600 cm^−1^,C–N str. at 1200–1020 cm^−1^, and C–H str. at 2960–2850 cm^−1^ [28]. It was found that there was no chemical interaction between sumatriptan succinate and excipients used as there were no changes in the characteristic peaks of sumatriptan succinate in the IR spectra of mixture of drug with excipients as compared to IR spectra of pure drug as shown in Figure 1.

**Figure 2:**
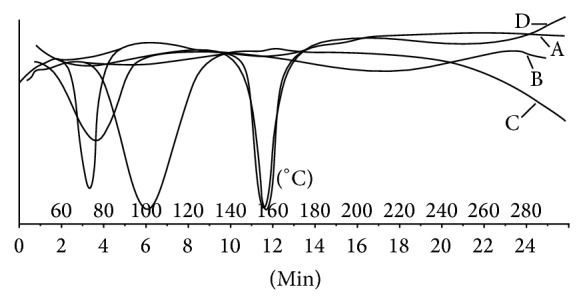
DSC spectra of (A) sumatriptan succinate, (B) polyox WSR205, (C) xanthan gum, and (D) optimized batch F8.

DSC as shown in Figure 2 indicated melting point of sumatriptan succinate, polyox WSR205, and xanthan gum in range of 159–162°C, 75–80°C, and 95–100°C, respectively. DSC spectra for optimized batch F8 showed melting point in range of 75–85°C which may be due to interlinking of both the polymers, whereas sumatriptan succinate showed sharp melting point at 159°C.

### 3.3. Preliminary Trial Batches for RRCT

It was observed that when concentration of microcrystalline cellulose varied from 12 to 61% of weight of RRCT, it had shown variation in the hardness of core tablet. As the drug release depends on the disintegration time, crospovidone was added as superdisintegrant by varying concentration from 1 to 10% to obtain optimized batch RRCT-4. Formulations (RRCT-4) containing crospovidone in higher concentration (10%) showed rapid drug release (92.45 ± 0.5%) in 16 seconds after coming in contact with dissolution fluid. As superdisintegrant swell giving burst effect which was need of the pulsatile drug delivery, so this formulation was finalized.

### 3.4. Preliminary Trial Batches for FPRT

As the total polymer weight in floating pulsatile layer of tablet was replaced with 100% of polyox WSR205, it had shown extended lag period of 8 ± 0.5 h which is higher than the predetermined lag time. This may be due to high value of hardness (>10 Kg/cm^2^). Hundred percent xanthan gum of total polymer weight in floating pulsatile layer of tablet showed lag period of 2 ± 0.5 h. This may be due to low value of hardness and compressibility. Both polymers alone failed to achieve predetermined lag period of 6-7 h. Therefore, to achieve lag period of 6-7 h, combination of polyox WSR205 and xanthan gum was used. Floating ability of FPRT was optimized to the ratio of 1 : 10 of citric acid and sodium bicarbonate, respectively. The batch with the ratio of 75 : 25 of polyox WSR205 and xanthan gum, respectively, showed lag period of 6 ± 1 h, floating lag time of 50 ± 10 sec, and hardness 5.5 ± 1 Kg/cm^2^. Further, this batch was used to carry out experimental design.

### 3.5. Precompression Characteristics of RRCT-4 and FPRT (F1–F9)

Precompression parameters for both powder blends of RRCT-4 and FPRT (F1–F9) showed results within specified limits. Results were expressed in the range for angle of repose (23–27°), bulk and tapped density (0.40–0.53 g/cm^2^), compressibility index (10–12), and Hausner's ratio (1-2). These values indicated that all the powder blends showed good flow property.

### 3.6. Postcompression Characteristics 

#### 3.6.1. RRCT-4

All postcompression parameter values, that is, thickness (0.63 ± 0.02 mm), diameter (0.24 ± 0.05 mm), uniformity of weight, (69.65 ± 0.08 mg), friability (0.71 ± 0.06%), drug content (99.3 ± 0.02%), hardness (2.53 ± 0.01 Kg/cm^2^), and disintegration time (16.5 ± 0.03 sec), were found within specified limits [21].

#### 3.6.2. FPRTs (F1–F9)

All factorial batches showed results for thickness (4.1 ± 0.2 mm), diameter (9.1 ± 0.1 mm), drug content (99.85 ± 0.2%), and tablet weight (323.87 ± 0.1 mg) which were within the specified range. But variation in polymer ratio drastically affected hardness and swelling index which further had major effect on buoyancy time, % drug release, and lag time as indicated in Table 4.

### 3.7. Swelling Index

**Figure 3:**
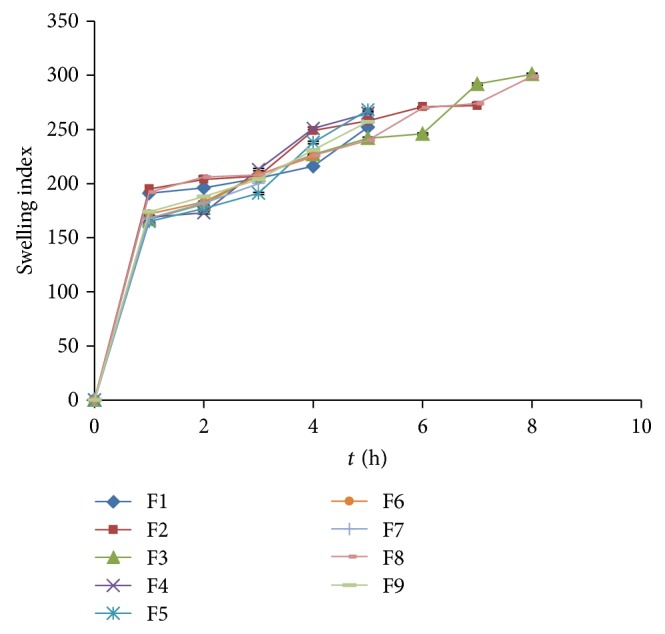
Swelling indices profile of factorial batches F1–F9 of polyox WSR205 and xanthan gum.

FPRTs consist of polyox WSR205 and xanthan gum in outer layer of the tablet. Polyether chains of polyox WSR205 formed hydrogen bonds with water and polymer tends to hydrate, forming superficial gel which eventually erodes as the polymer dissolves. Xanthan gum has high degree of swelling capacity involving water uptake and small degree of erosion due to polymer relaxation. Therefore, direct correlation between swelling and lag time was observed. It was found that the formulations having maximum swelling indices showed higher lag time. Once the tablet gets burst, the swelling index slowly decreases due to erosion of polymers which depend on the individual tablet burst time. Swelling indices profile of factorial batches F1–F9 is as shown in Figure 3.

### 3.8. *In Vitro* Drug Release (Lag Time) Study

**Figure 4:**
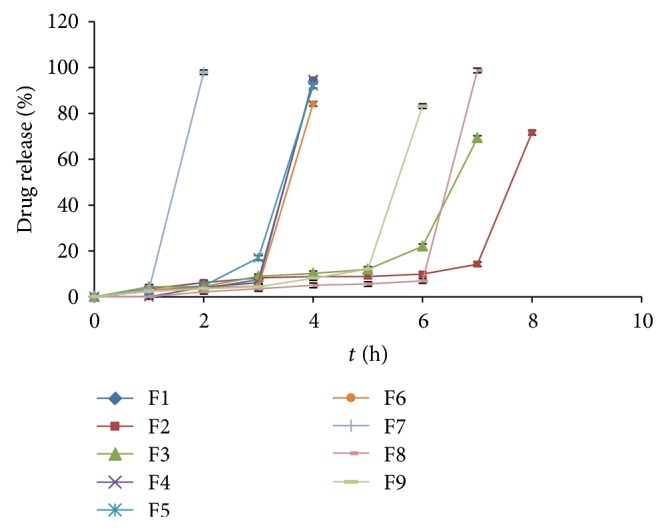
Dissolution profile of factorial batches F1–F9 of polyox WSR205 and xanthan gum.

As the coated tablet was placed in the dissolution medium, the hydrophilic polymeric layer starts to swell, which then underwent progressive modification in terms of thickness and consistency. After swelling up to a limiting thickness, the outer shell was ruptured under the pressure applied by the swelling of the tablet and drug gets released. The drug release profiles relevant to the coated tablet showed that a lag phase was followed by quick delivery of drug due to the core containing superdisintegrant. Both the polymer polyox WSR205 and xanthan gum have swelling as well as erosion property, due to which polymer alone and combinations showed different drug release profiles. Formulations (F1–F9) coated with different concentration of polyox WSR205 and xanthan gum as per the factorial design have given the lag time varying from 2 to 6 h as shown in Figure 4. Formulation F8 showed lag period of 7 h with drug release of 98.69% ± 2% which was considered to be suitable for chronotherapeutic objective.

### 3.9. Kinetics of the Drug Release Data

Based on the* R*-value, best fit model was selected [29]. F1 showed Hixson Crowell as best fit model which indicated the constant release from the system where there is a change in surface area and diameter of tablets. Factorial batches F2–F9 showed Korsmeyer-Peppas as best fit model. The formulations prepared primarily contain polymers which undergo Fickian diffusion and/or polymer relaxation process. So, in this case, the system can be declared following zero order only, if the value of release exponent or “*n*” comes out as equal to or greater than the threshold value, that is, 0.89.  Table 5 indicates statistical analysis of kinetics data of drug release for factorial batches F1–F9.

### 3.10. Response Surface Plots

Response surface methodology has been used as experimental design to determine the effect of independent variables on all possible dependent variables. Experimental design was applied for variable concentration of polymers to find out effect on hardness, swelling index, floating lag time, burst time, and drug release. As concentration of effervescent agents and superdisintegrant was previously optimized, application of experimental design had shown nonsignificant effect on formulations with respect to floating lag time, burst time, and drug release. Effect of independent variables on hardness and swelling index is as shown in equation (2). Consider

(2) Hardness =+6.15+8.843E−003A+0.40B−0.24ABSwelling  index=+227.72+0.23A+4.57B−4.81AB,

where *A* indicates polyox WSR205 and *B* indicates Xanthan gum.

**Figure 5:**
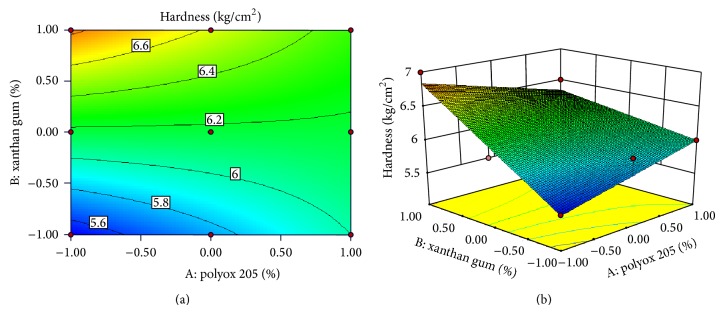
Contour plot and response surface plot showed relationship in between hardness and % of polymer.

**Figure 6:**
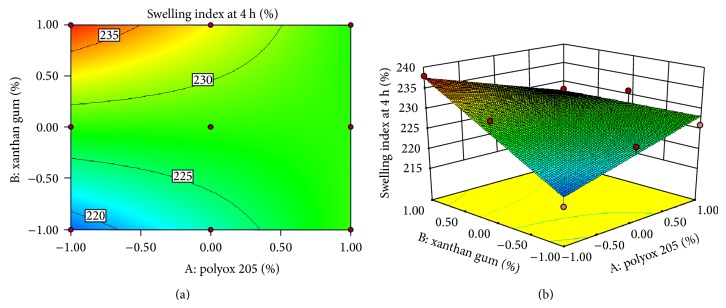
Contour plot and response surface plot showed relationship in between swelling index and % of polymer.

Both the polymer individually had shown their effect on hardness as well as swelling index, but combined effect of the polymers was null on both responses. Polyox WSR205 showed greater linear effect on hardness, while xanthan gum showed greater linear effect on swelling index as it has high degree of swelling capacity. Values of “Prob >* F*” (*P* value) less than 0.0500 indicate model terms were significant. The model obtained was 2FI Model. The Model* F*-value of 7.95 implies the model is significant. “Adeq Precision” measures the signal to noise ratio. A ratio greater than 4 is desirable. Obtained ratio was 8.084 and 9.077 indicating an adequate signal. There is only a 2.38% chance that a “Model* F*-Value” this large could occur due to noise. This receives confirmation from the mathematical model (ANOVA) generated for responses as indicated in Table 6. The response surface plots and contour plots showing the effect of polymer ratio on hardness and swelling index are as indicated in Figures 5 and 6, respectively.

### 3.11. *In Vivo* X-Ray Study

**Figure 7:**
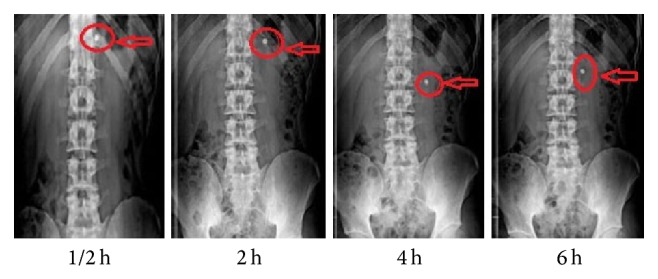
X-ray graph of optimized batch F8 (polyox WSR205 and xanthan gum).

The duration of tablet in stomach and upper part of intestine was monitored by radiograms. The radiographs taken after 0.5 h indicated buoyancy of the tablet, in case of volunteers in fed state as shown in Figure 7. The tablet did not sink in stomach nor it adhered to stomach mucosa during the time of retention in stomach. It remains in floating state for 6 hours as can be seen in Figure 7. It was observed that the tablet stayed in stomach for 6 ± 0.5 h for optimized batch F8 indicating successful gastric retention ability.

### 3.12. Stability Study

Accelerated stability study indicated that the optimized formulation F8 was physically as well as chemically stable after 3 months.

### 3.13. Validation of Statistical Model

The predicted responses of the optimized batch F8 (this batch gave required lag time of 7 h and so was considered for comparison) and corresponding actual experimentally observed values were found to be close as indicated in Table 7. Thus, it was found that the model developed to predict responses was statistically significant.

### 3.14. Similarity Factor Study

Similarity factor between marketed rapid release tablet of sumatriptan succinate (Suminat 50; Sun Pharma. Ind. Ltd.) was compared with optimized formulation as test product. The study involves data obtained from the drug release. Factor *f*_2_ obtained was 12 which is less than 50 confirming no similarity in drug release of both test and marketed formulation. This is because marketed formulation was rapid release, while the optimized formulation was burst release after lag time.

## 4. Conclusion

Migraine chronotherapy based floating pulsatile press coated tablet of sumatriptan succinate was successfully developed. Optimized FPRT batch F8 showed lag time of 7 ± 0.1 h and 55 ± 2 sec floating lag time along with maximum drug release (98.69 ± 2%).* In vivo* X-ray study indicated that FPRT may increase gastric residence time due to effervescent agents citric acid and sodium bicarbonate in proportion of 1 : 10. The optimized FPRT-8 may be used for the administration at bed time which will release sumatriptan succinate in the early morning when chances of migraine attacks are more. However, there is further need of investigation for clinical acceptance of this novel drug delivery system.

## Figures and Tables

**Table 1:** Formulation of RRCT.

Ingredients	CT1	CT2	CT3	CT4
Sumatriptan succinate	25	25	25	25
Crospovidone	1	3.5	1.4	7
Magnesium stearate	3	3	1	1
Microcrystalline cellulose	8	37.5	42.6	37
Talc	—	1	—	—
Lactose	33	—	—	—

Total weight (mg)	70	70	70	70

All weights are in mg.

**Table 2:** Variables and coded levels data.

Variables used	Coded levels		
	−1	0	1
Polyox WSR205 (mg)	157.5	167.5	180
Xanthan gum (mg)	45	57.5	67.5

**Table 3:** Formulation of floating pulsatile tablet.

Batch code	Coded levels		Polyox WSR205 (mg)	Xanthan gum (mg)	Citric acid (mg)	Sodium bicarbonate (mg)	Dicalcium phosphate (mg)	Core tablet (mg)
	Variable 1 (Polyox WSR205)	Variable 2 (Xanthan gum)						
F1	−1	−1	157.5	45	0.46	4.6	47.44	70
F2	1	0	180	67.5	0.46	4.6	2.44	70
F3	0	0	167.5	57.5	0.46	4.6	24.94	70
F4	−1	0	157.5	57.5	0.46	4.6	34.94	70
F5	−1	1	157.5	67.5	0.46	4.6	24.94	70
F6	0	−1	167.5	45	0.46	4.6	37.44	70
F7	0	1	167.5	67.5	0.46	4.6	14.94	70
F8	1	−1	180	45	0.46	4.6	24.94	70
F9	1	1	180	57.5	0.46	4.6	12.44	70

Total weight of Tablet = 325 mg.

**Table 4:** Evaluation of FPRTs.

Batch code	Hardness (Kg/cm^2^) *N* = 6	Buoyancy time (sec) *N* = 6	Drug release after lag time (%) *N* = 6	Lag time (h) *N* = 6
F1	5.5 ± 0.1	45 ± 3	93.77 ± 2	4 ± 0.5
F2	6.5 ± 0.2	900 ± 6	71.56 ± 3	8 ± 0.1
F3	6 ± 0.1	33 ± 5	69.21 ± 5	7 ± 0.1
F4	6 ± 0.2	40 ± 4	94.71 ± 2	4 ± 0.3
F5	7 ± 0.2	900 ± 8	92 ± 3	4 ± 0.4
F6	6 ± 0.1	35 ± 2	84.1 ± 4	4 ± 0.3
F7	6.5 ± 0.1	60 ± 4	97.87 ± 1	2 ± 0.5
F8	6 ± 0.1	55 ± 2	98.69 ± 2	7 ± 0.1
F9	6 ± 0.1	50 ± 3	83.15 ± 3	6 ± 0.1

*N* = 6; values are expressed in mean ± SD (standard deviation).

**Table 5:** Statistical analysis of kinetics data of drug release.

Batch code	Zero order	1st order	Matrix	Korsmeyer-Peppas			Hixson Crowell	Best fit model
	*R*	*R*	*R*	*R*	*n*	*k*	*R*	
F1	0.9994	0.9996	0.9724	0.9756	0.6881	30.0459	0.9998	Hixson Crowell
F2	0.7084	0.6532	0.5837	0.8490	1.0365	2.3164	0.6711	Korsmeyer-Peppas
F3	0.8265	0.7953	0.6804	0.8829	1.4226	2.0525	0.8054	Korsmeyer-Peppas
F4	0.7003	0.6777	0.5547	0.8764	2.5080	0.8631	0.6845	Korsmeyer-Peppas
F5	0.7920	0.7478	0.6423	0.9495	2.4981	1.2806	0.7621	Korsmeyer-Peppas
F6	0.6859	0.6679	0.5421	0.7683	2.1137	1.2038	0.6732	Korsmeyer-Peppas
F7	0.8535	0.8460	0.7275	1.000	4.9821	2.3469	0.8483	Korsmeyer-Peppas
F8	0.5790	0.5419	0.4472	0.7728	1.4752	0.9012	0.5527	Korsmeyer-Peppas
F9	0.6776	0.6316	0.5335	0.8806	1.7938	0.9048	0.6460	Korsmeyer-Peppas

**Table 6:** ANOVA used to generate statistical models.

Response model	Sum of squares	df.	Mean square	*F* value	*P* value	*R* ^2^	Adequate precision
Hardness	1.22	3	0.41	7.32	0.0281	0.8145	0.084
Swelling index at 4 h	228.89	3	76.30	7.95	0.0238	0.8267	9.077

**Table 7:** Comparisons of predicted values and observed values.

Polymer	Coded level	Actual level	Responses	Hardness	Swelling index
			Predicted values	6.002	228.18
			Observed values	6	226
Polyox WSR205	1	180	Standard deviation	0.2359	3.098
Xanthan gum	−1	45	Standard error mean	0.2049	2.6919

## Abbreviations

WSR: Water soluble resin
UV: Ultra violet
LOD: Limit of detection
LOQ: Limit of quantitation
FTIR: Fourier transform infrared
DSC: Differential scanning spectroscopy
KBr: Potassium bromide
HCl: Hydrochloric acid
USP: United States Pharmacopoeia
ICH: International Conference On Harmonization
RH: Relative humidity
SD: Standard deviation
RRCT: Rapid release core tablet
CT: Core tablet
FPRT: Floating pulsatile release tablet
Str.: Stretching
Df.: Difference
ANOVA: Analysis of variance
Def.: Deformation
h: Hour
rpm: Rotations per minute.
